# Lactylation and its roles in diseases: a systematic bibliometric exploration of the research landscape

**DOI:** 10.3389/fonc.2025.1569651

**Published:** 2025-07-02

**Authors:** Mengjia Li, Yuhang Liu, Chuhan Jiang, Shiyu Zhang, Mengyu Nan, Jiaxi Liu, Yangyang Han

**Affiliations:** ^1^ Department of Biology, School of Basic Medical Sciences Xinjiang Medical University, Urumqi, China; ^2^ Xinjiang Key Laboratory of Molecular Biology for Endemic Diseases, Xinjiang Medical University, Urumqi, China

**Keywords:** lactylation (Kla), histone, cancer, CiteSpace, VOSviewer, bibliometric analysis

## Abstract

**Background:**

Lactylation, a post-translational modification (PTM), has gained attention for its role in disease pathogenesis, particularly in cancer and immune regulation. Initially viewed as a glycolysis byproduct, lactate is now recognized as a precursor for histone lysine lactylation (Kla), which regulates gene transcription and epigenetic processes. Dysregulation of lactylation is linked to malignancies, inflammation, and metabolic diseases. Despite growing research, a systematic bibliometric analysis of lactylation remains absent. This study addresses this gap by analyzing lactylation research from 2019 to 2024, focusing on its disease-related mechanisms and therapeutic potential.

**Method:**

Data were extracted from the Web of Science Core Collection (2019–2024), yielding 198 relevant articles after screening. Bibliometric analysis was conducted using Microsoft Excel, VOSviewer, Scimago Graphica and CiteSpace. Excel tracked publication trends, VOSviewer generated author density maps, and CiteSpace visualized collaboration networks, co-cited references, and keyword clusters. Scimago Graphica generated Map of country cooperation networks. The study identified research trends, collaborative patterns, and emerging hotspots in lactylation research.

**Result:**

Lactylation research has surged exponentially since 2022, with China as the primary contributor (92.42% of publications). Dominant keywords converge on lactylation’s role as a direct epigenetic regulator of gene activation, enabling transcriptional reprogramming in diseases; Lactylation drives bladder cancer progression via immunosuppressive genes, mediates myoblast differentiation for muscle repair, and disrupts signaling through non-histone targets; Emerging focus on “differentiation” and “metabolic regulation” highlights its potential as a cellular reprogramming target, crucial for advancing regenerative medicine and combating inflammation-linked pathologies.

**Conclusion:**

This study provides the first bibliometric analysis of lactylation research, highlighting its rapid growth and global significance. China leads the field, with extensive contributions from its institutions. Lactylation’s role in disease mechanisms, particularly cancer and immune regulation, underscores its therapeutic potential. Emerging research on differentiation and metabolic regulation offers new directions for future studies. Further investigation into lactylation’s molecular mechanisms and therapeutic applications is essential for advancing disease diagnosis and treatment.

## 1 Introduction

**Figure 1:**
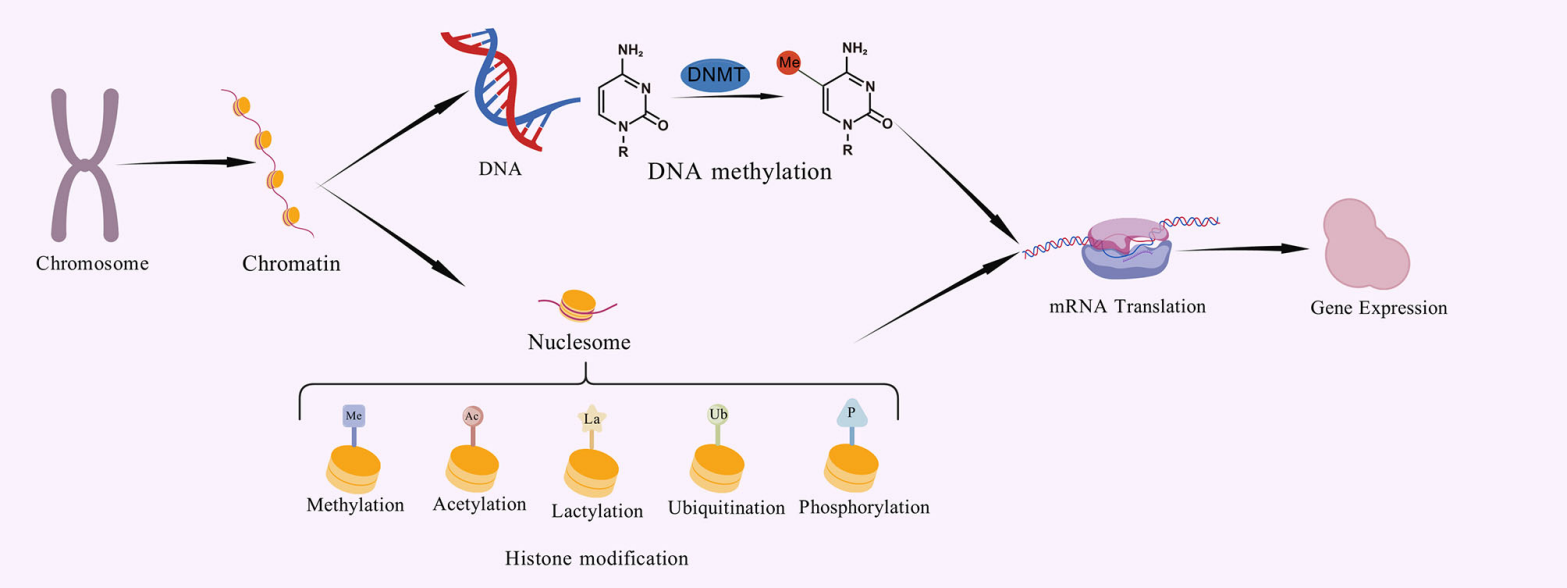
These modifications are essential for cell differentiation, development and adaptation to the environment, and can alter chromatin structure or provide signalling markers that determine whether a gene can be transcribed, allowing precise control of gene expression without altering the DNA.

Metabolic reprogramming is a central feature of numerous diseases such as cancer, inflammation, and infection, and its by-product lactate was once regarded as a pure metabolic waste product (1). However, the disruptive role of lactate as a key signaling molecule is being redefined - directly reshaping the chromatin landscape and gene-expression programs by driving novel histone post-translational modifications known as “lactylation,” opening new perspectives on understanding the central role of metabolic-epigenetic interactions in pathophysiology. Elucidating the precise regulatory mechanism of lactylation in specific biological processes has become a frontier hotspot to fill major knowledge gaps and reveal potential therapeutic targets. Epigenetic regulation - reversible mechanisms that do not alter DNA sequence but genetically affect gene function - is key to cell adaptation to the environment and fate. Among them, histone post-translational modifications (PTMs) constitute a complex and precise “epigenetic code” (2, 3). (**Figure 1**) In recent years, lactylation has been revealed as a new type of PTM on lysine residues directly powered by lactic acid, which has revolutionized our understanding of the functional boundaries of metabolites. Zhang et al. used mass spectrometry in 2019 to find the first evidence of a mass shift, measuring 72.021Da, on lysine residues in human breast cancer cells (4). Lactylation modification has unique molecular features and potential biological functions, and has rapidly become a key bridge connecting the metabolic state of cells with epigenetic remodeling.

**Figure 2:**
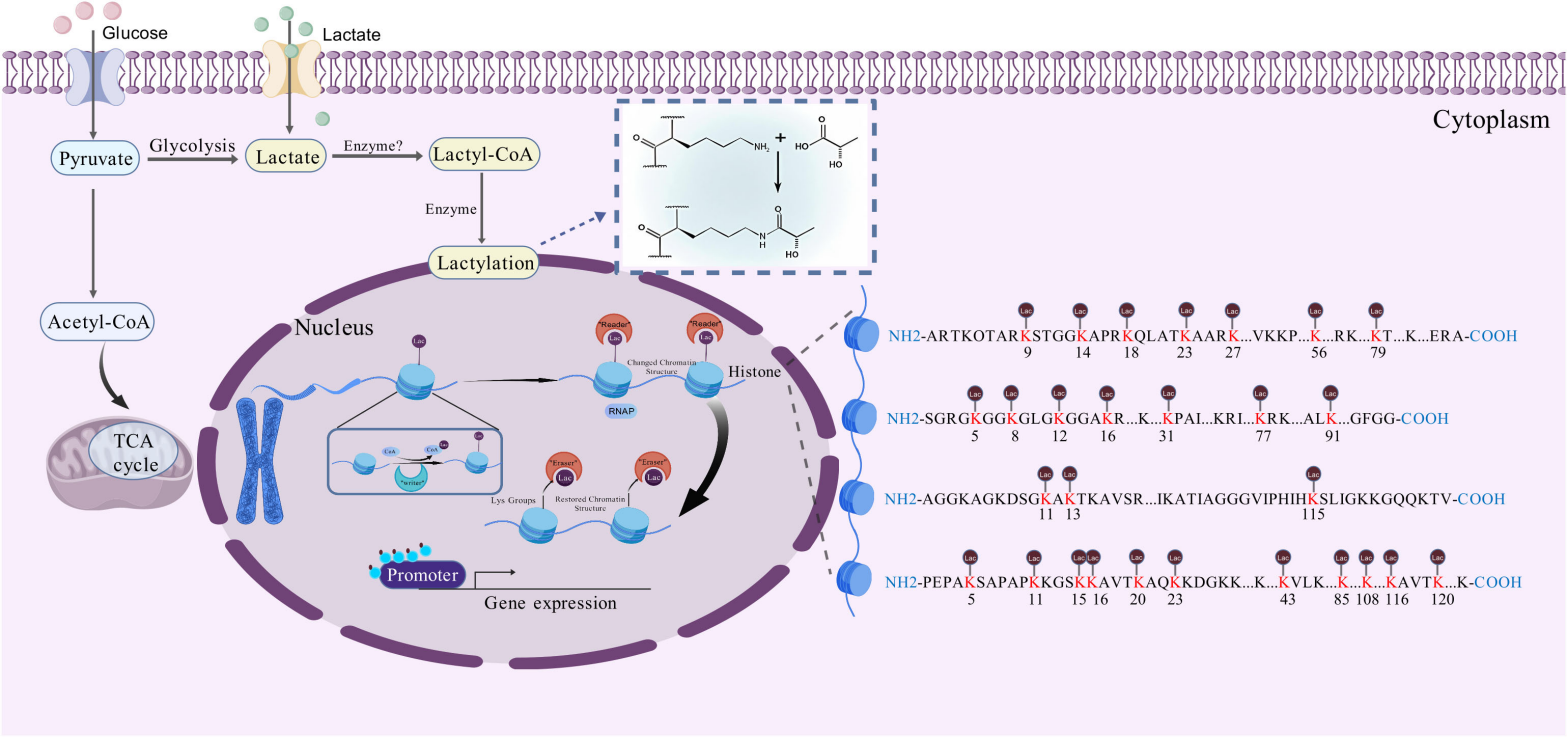
The specific regulatory process of lactylation modification.In terms of other histone modifications, the process of histone lactylation may proceed as follows. Firstly, lactate from multiple pathways combines with intracellular CoA to form lac-CoA. Next, lac-CoA enters the nucleus and, under the action of an acyltransferase known as a ‘writer’, transfers the lactate acyl group to a lysine site. After undergoing modification, the chromatin structure will change. With the so-called ‘Reader’, the information contained in these structural changes will be identified, leading to changes in the levels of transcription of downstream genes and, consequently, variations in gene expression, protein function and cell phenotype. Once the modification process is complete, the ‘Eraser’ enzyme recognises the lactate acyl group and unbinds it from the lysine site, restoring the chromatin structure to its original state. The restored lysine sites can then be used for other HPTMs. At this point, the lactylation modification process is complete.

The importance of lactylation modification in diseases has emerged: its abnormal dynamics have been confirmed to be closely related to key pathological processes such as macrophage immune polarization, tumor microenvironment shaping, neuroinflammatory response, and pathogen infection process (5). For example, Kla is highly associated with the development of malignancies and has been found in macrophages, immune-related cells, non-small cell lung cancer (NSCLC), and ocular melanoma (6). Histone lactylation plays a key role in driving tumor growth and is a useful diagnostic marker for a variety of malignancies including colorectal cancer (7), hepatocellular carcinoma (HCC) (6), and ocular melanoma (8). These findings strongly imply that lactylation is a central effector mediating metabolic dysregulation to trigger pathological gene expression programs. The specific regulatory process of lactylation modification is shown in **Figure 2**. Direct binding of lactate to free amine groups on histone lysine residues is a non-spontaneous chemical reaction under physiological conditions. One proposed enzymatic mechanism suggests that lactate is first activated to lactyl-CoA, which is then transferred to histones by a specific “writer” enzyme (9). However, lactyl-CoA has not been robustly detected in biological samples and is found at markedly lower concentrations (25 to 350-fold lower) than other acyl-CoAs, such as acetyl-CoA, succinyl-CoA, and propionyl-CoA (10). The currently known histone Kla writer is HAT p300, it could add the lactyl group from lactyl-CoA to certain lysine sites by acting as a catalyst (11). They also discovered that KAT8, a pan-Kla writer lysine acetyltransferase, is the enzyme that installs Kla on a wide variety of protein substrates involved in various biological processes. The development of colorectal cancer tumors was suppressed by KAT8 deletion, particularly in a high-lactic tumor microenvironment (12). In contrast, HDAC1–3 and SIRT1–3 can function as delactylases to cleave the Kla marks (13). Additionally, two molecular probes, p-H4K16la-Alk and p-H4K16la-NBD, were created by Fan et al. To identify possible erasers of Kla from cell lysates and to directly detect the eraser-Kla delactylation processes using fluorescence indication (14). Furthermore, YiaC can function as a writer to catalyze the addition of Kla, whereas CobB can function as an eraser to reverse this modification in E. coli (15). Alternatively, a non-enzymatic mechanism proposes that methylglyoxal (MGO), a glycolytic byproduct, reacts with glutathione to form S-lactoylglutathione. This intermediate can directly lactylate lysine residues via non-enzymatic nucleophilic acyl-substitution reactions (16). In summary, despite its growing importance, the dynamic map of lactylation modification in specific cell types or physiological/pathological pathways, the precise target gene profile, the upstream regulatory enzymes (“ writers “/” erasers “), and the downstream functional implications of lactylation modification remain largely unexplored.

Through bibliometric analysis and review, this study aims to identify research trends, systematically assess the quality of research published in relevant journals, and understand the research progress of different disciplines, countries, institutions and authors. Through the intuitive mapping, we can predict the future direction of this field, and point out the next research direction for researchers. Through this review, we will deeply analyze the core mechanism of lactylation modification in diseases, and clarify its functional regulation on epigenetic modification and its contribution in related diseases.

## 2 Methodology

### 2.1 Data collection

**Figure 3:**
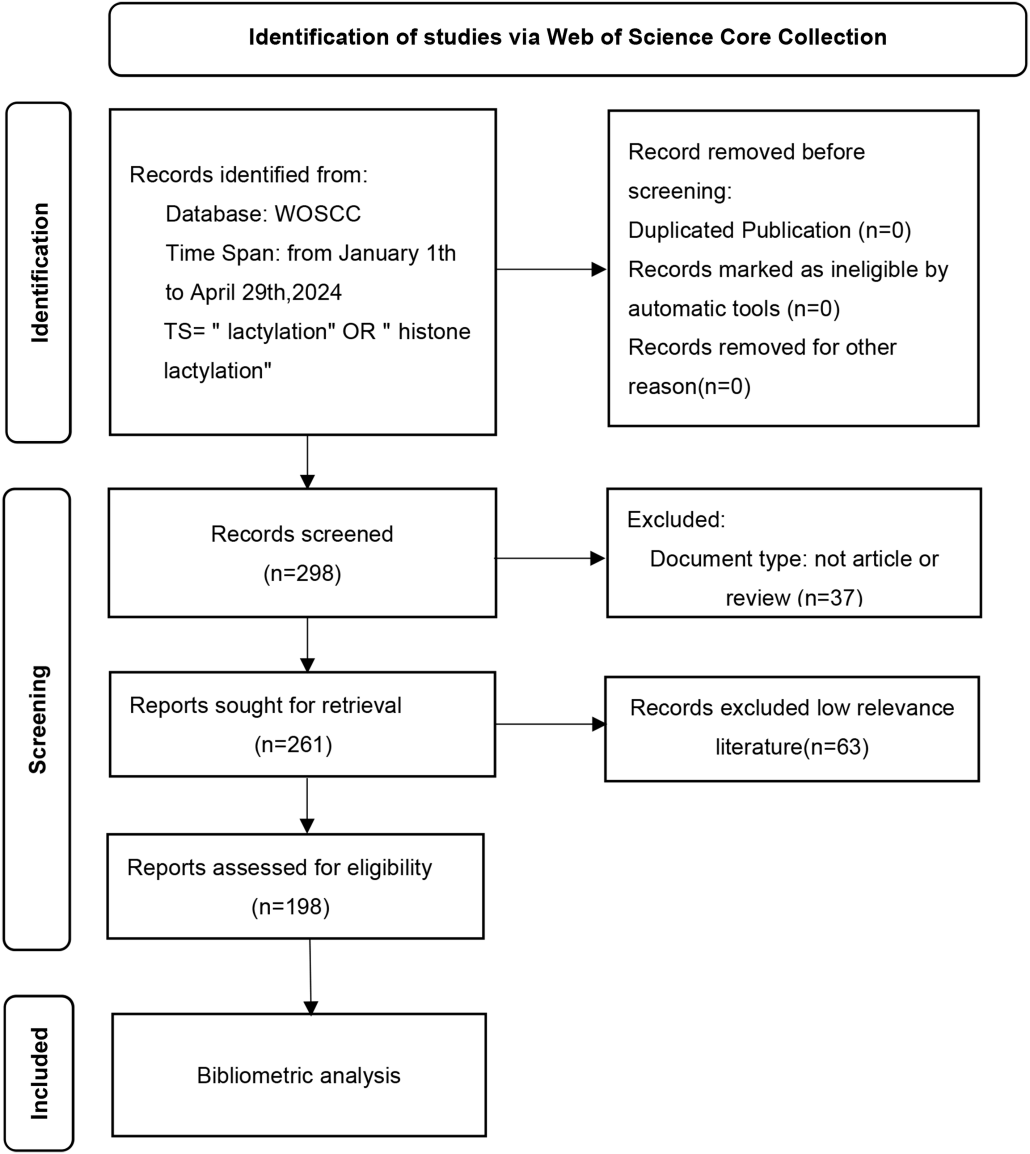
A study flow diagram of lactylation in cancers from WoSCC. WoSCC, web of science core collection.

The Web of Science Core Collection database was used to extract the raw data. The search formula was set as follows: Lactylation (All field) and 2019-2024 (Year Published) and Review or Review Article (Document Types). A total of 261 unique records were identified. After our selection of the papers, the number of papers remaining was 198 (**Figure 3**). Documents were all saved as records and references were output in the form of all records and references, saved as plain text and stored in Download txt format. To guarantee the precision and reliability of the data, the entire data retrieval and collection process was completed on 5 May 2024.

### 2.2 Data analysis and visualization

All statistical analyses were performed using Microsoft Excel Office 365, VOSviewer version 1.6.20, Scimago Graphica 1.0.50 and CiteSpace 6.3.R1. Microsoft Excel Office 365 was employed to construct a chart illustrating the fluctuations in the volume of documents issued on an annual basis within this field. VOSviewer is a software tool for constructing and visualizing bibliometric networks, developed by Leiden University in the Netherlands. The software generates maps of any type of text and performs a range of analyses, including publication statistics, cooperative network analysis, co-occurrence analysis and document co-citation analysis. In this study, VOSviewer has been used for the creation of an author density map.

CiteSpace is an open-source Java application that helps researchers understand their field through analysis and visualization of citation networks, trends and patterns. In this study, journals, countries, institutions, co-cited references and keywords were explored using CiteSpace to generate visualized maps.

Scimago Graphica is a web application developed by Scimago Lab that focuses on visualizing scientific research patterns through geospatial maps. The tool combines bibliometric indicators with geographic coordinates to support the analysis of research performance and collaboration patterns across regions and institutions. In this study, it was used to generate geospatially overlaid maps to visualize the global distribution and collaborative networks of research activities in relevant fields., 

## 3 Results

### 3.1 Trends in publication outputs

**Figure 4:**
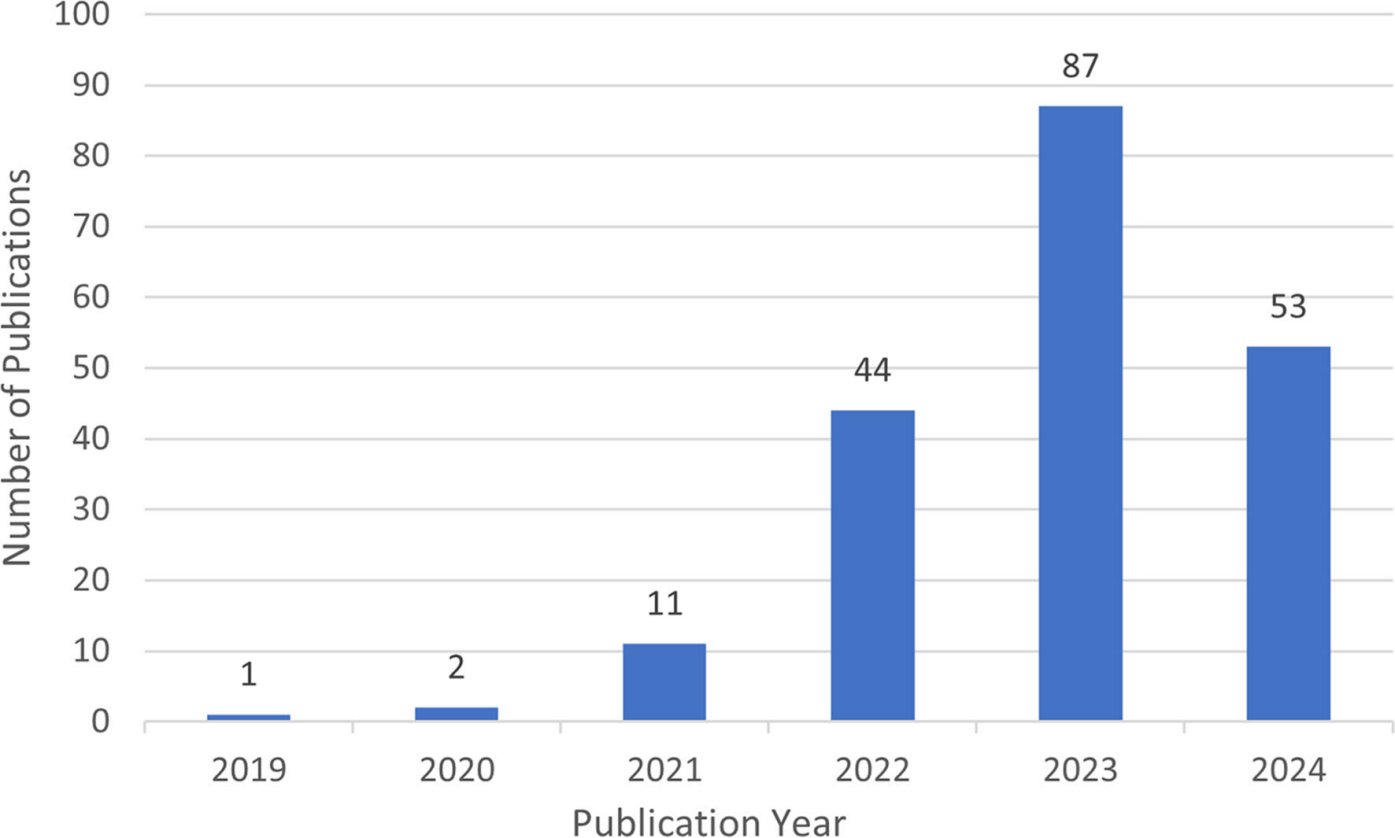
The number of publications from 2019 to 2024 in the field of lactylation.

The general trend of research activity in the field is reflected by the number of publications in different time periods. The number of articles on lactylation shows a marked increase on an annual basis, as shown in **Figure 4**. In 2019, when the concept of lactylation was proposed firstly (Zhang). Until 2021, there are 14 filed articles on lactylation, which means that research in this filed is still in its infancy. From 2022 to 2024 publications are growing exponentially, indicating that lactylation research continues to explode. This shows that the research on lactylation has entered a phase of rapid development in the last few years. The results of the prediction analysis indicate that the number of papers published in 2024 will exceed 356.

In addition, a total of 198 relevant studies in the field of lactylation research were collected from the Web of Science core collection between 2019 and 2024. Regarding the time of publication, lactylation was first introduced between 2019 and 2020, which meant that there needed to be literature output on related topics during this period. The 2021 research phase has now come to an end. The project has entered a phase of accelerated development, accompanied by a significant increase in the number of publications.

### 3.2 Country/region and institution analysis

**Figure 5:**
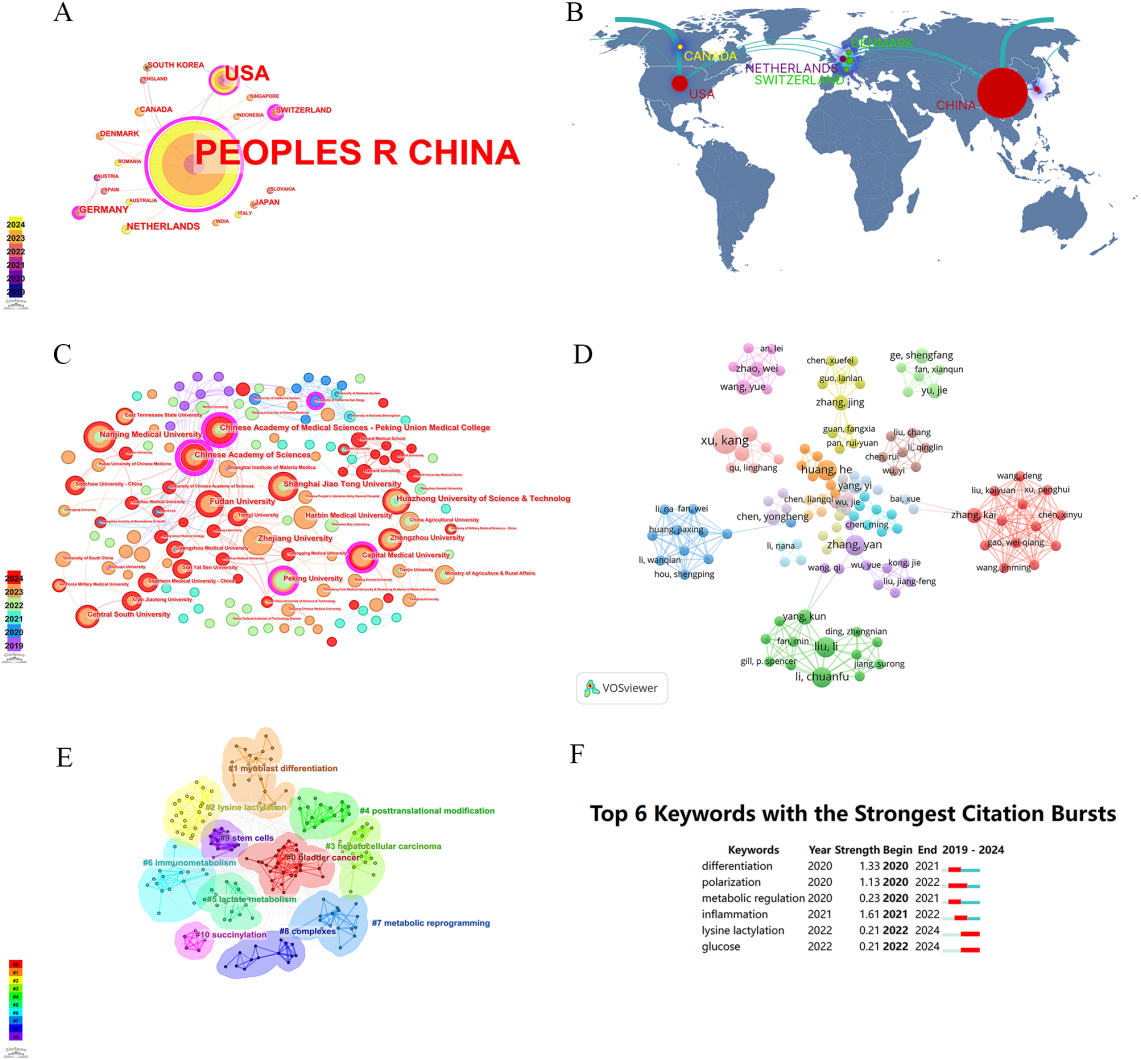
Analysis of lactylation biomarker-related data. **(A)** Visualization of collaboration networks among countries/regions using CiteSpace. Nodes of varying colors represent different clusters of countries/regions. **(B)** Countries/regions engaged in lactylation-related biomarker research. The connections between countries demonstrate their collaboration and interconnection. **(C)** Visualisation of inter-agency collaborative networks using CiteSpace(Colours represent the number of publications per year). **(D)** The visual picture of authors in the field of lactylation.Their interconnections represent co-operation. **(E)** The visual picture of keywords (Each color represents a sort of keywords). **(F)** The top 6 outbreaks (The length of the red line represents the time of occurrence of emergent word).

The first publication related to lactylation was introduced in 2019 by Zhang, representing the sole article on the subject at that time. They are leading the way in research carried out by scientists across the globe. All eligible publications from 19 countries/regions, of these, the production of the People’s Republic of China was by far the most important with 183 documents, followed by the United States (17). An insight into trends and patterns in document production is provided by analyzing changes in the annual volume of documents issued by the ten most productive countries/regions. A horizontal comparison of the two countries reveals that China has published more research annually than the United States. Countries such as Germany, the Netherlands, Switzerland and Denmark have a lower total research output than China and the United States. However, there has been a significant increase in research activity in recent years. It is evident that the close and amicable collaboration between disparate nations constitutes a pivotal rationale for this phenomenon. An examination of the CiteSpace network visualization map of countries and regions revealed that there is a notable degree of intercountry cooperation, which has considerably influenced the search trend for lactylation. The countries indicated by purple circles, including the People’s Republic of China (0.83), Switzerland (0.43) and Germany (0.17), exhibited the highest betweenness centrality, which signifies their pivotal role in international collaboration (**Table 1**). We also used Scimago Graphica to make a map depicting co-operation with other countries. With **Figure 5A** and **Figure 5B**, we can clearly observe that The People’s Republic of China had the highest betweenness centrality, and the countries/regions that co-operated most frequently with the People’s Republic of China were the United States, South Korea and Switzerland. Meanwhile, The United States also maintains relatively close cooperation with the Netherlands, Switzerland, Canada and South Korea. We constructed a network visualization map of publications from CiteSpace (**Figure 5C**) to explore the productive institutions in this field. The ten most productive institutions in relevant research are shown in **Table 2**. The leading institutions were the Chinese Academy of Sciences (12, 6.06%), Shanghai Jiao Tong University (11, 5.55%), Nanjing Medical University (11, 5.55%), Chinese Academy of Medical Sciences - Peking Union Medical College (10, 5.05%) and Zhejiang University (10, 5.05%). The **Figure 5B** clearly shows the collaboration between institutions. China accounts for 100% of the top ten most productive institutions. In summary, China’s research in lactylation maintains a dominant position.

**Table 1:** The 10 most productive country in the field of Lactylation (including the number of documents, the percentage of total literature and the centrality).

Rank	Country	Year	Document	Percentage (n/198)	Centrality
1	China	2019	183	92.42	0.83
2	USA	2019	19	9.59	0.11
3	Germany	2021	3	1.51	0.17
4	Netherlands	2022	3	1.51	0
5	Canada	2023	2	1.01	0
6	Denmark	2022	2	1.01	0
7	Japan	2021	2	1.01	0
8	South Korea	2019	2	1.01	0
9	Switzerland	2022	2	1.01	0.43
10	Australia	2024	1	0.50	0

**Table 2:** The 10 most productive institutions in the field of Lactylation.

Rank	Institution	Articles(n)	Percentage(n/198)	Location
1	Chinese Academy of Sciences	12	6.06	China
2	Shanghai Jiao Tong Univ	11	5.55	China
3	Nanjing Medical Univ	11	5.55	China
4	Chinese Academy of Medical Sciences - Peking Union Medical College	10	5.05	China
5	Zhejiang Univ	10	5.05	China
6	Fudan Univ	9	4.54	China
7	Huazhong Univ of Science & Technology	9	4.54	China
8	Zhengzhou Univ	8	4.04	China
9	Capital Medical Univ	8	4.04	China
10	Central South Univ	7	3.53	China

### 3.3 Analysis of authors

The researchers have co-authored publications related to lactylation modifications, and the top 10 most cited journals and authors are listed in the **Table 3** below. Zhang D’s 2019 article Metabolic regulation of gene expression by histone lactylation published in NATURE was the most cited with 192 citations.

**Table 3:** The top 10 journals that contribute to this filed (Including citations, count and IF (2023)).

Rank	Journal	Count	Citations	IF (2023)
1	Frontiers in Immunology	11	93	5.7
2	International Journal of Biological Sciences	5	45	8.2
3	Science Advances	5	88	11.7
4	Cell Reports	4	119	7.5
5	Frontiers in Cell and Developmental Biology	4	42	4.6
6	Journal of Translational Medicine	3	12	6.1
7	Advanced Science	3	14	14.3
8	Frontiers in Genetics	3	23	2.8
9	Cell Metabolism	3	129	27.7
10	Cancer Letters	3	47	9.1

Analysis of authors can reflect active authors in lactylation modification studies (**Figure 5D**). VOSviewer is used to create an author map, where nodes represent authors (with larger circles indicating more publications) and lines between nodes indicate the degree of collaboration between two authors on an article.

### 3.4 Analysis of references

In bibliometric analysis, the metrics for judging the impact of an article include the number of citations and the impact factor of the article. As of May 2024, the top ten most influential references were analyzed out of the 198 relevant literatures obtained from the search. The results of this analysis are shown in **Table 4**. Among them, the most cited article is “Metabolic regulation of gene expression by histone lactylation” (n=186) published in 2019. This article is pioneering in its definition of lactate, a conventionally underappreciated byproduct of metabolism, as an epigenetic regulatory molecule, thereby inaugurating a novel research domain at the intersection of “metabolism and epigenetics.” Furthermore, the total citations (TC) per year were calculated, with the highest figure being published in 2021 by Yu J et al. The study, titled “Histone lactylation drives oncogenesis by facilitating m6A reader protein YTHDF2 expression in ocular melanoma” (n=38.3), has garnered significant attention in the research community. This study elucidates the oncogenic function of histone lactylation in tumorigenesis. The present study focuses on ocular melanoma and illustrates that lactylation levels are positively correlated with tumor progression. The elevated TC per year indicates a substantial number of subsequent studies that have focused on the role of lactylation in tumorigenesis and progression. This provides a theoretical foundation for targeting metabolic-epigenetic pathways as a treatment for tumors.

**Table 4:** Top 10 Influential Literature in the field of Lactylation.

Ranking	Total citations	TC per year	Year	First author	Journal	Title (DOI)	IF (2023)
1	186	37.2	2019	Zhang D	NATURE	Metabolic regulation of gene expression by histone lactylation (10.1038/s41586-019-1678-1)	50.5
2	115	38.3	2021	Yu J	GENOME BIOLOGY	Histone lactylation drives oncogenesis by facilitating m6A reader protein YTHDF2 expression in ocular melanoma (10.1186/s13059-021-02308-z)	10.1
3	71	35.3	2022	Yang K	CELL DEATH AND DIFFERENTIATION	Lactate promotes macrophage HMGB1 lactylation, acetylation, and exosomal release in polymicrobial sepsis (10.1038/s41418-021-00841-9)	13.7
4	68	17	2020	Irizarry-Caro RA	PNAS	TLR signaling adapter BCAP regulates inflammatory to reparatory macrophage transition by promoting histone lactylation (10.1073/pnas.2009778117)	9.4
5	57	28.5	2022	Xiong J	MOLECULAR CELL	Lactylation-driven METTL3-mediated RNA m6A modification promotes immunosuppression of tumor-infiltrating myeloid cells (10.1016/j.molcel.2022.02.033)	14.5
6	54	13.5	2020	Li LP	NATURE METABOLISM	Glis1 facilitates induction of pluripotency via an epigenome-metabolome-epigenome signalling cascade (10.1038/s42255-020-0267-9)	19.2
7	53	17.7	2021	Hagihara H	CELL REPORTS	Protein lactylation induced by neural excitation (10.1016/j.celrep.2021.109820)	7.5
8	53	26.5	2022	Moreno-Yruela C	SCIENCE ADVANCES	Class I histone deacetylases (HDAC1-3) are histone lysine delactylases (10.1126/sciadv.abi6696)	11.7
9	52	17.3	2021	Cui HC	AM J RESP CELL MOL	Lung Myofibroblasts Promote Macrophage Profibrotic Activity through Lactate-induced Histone Lactylation (10.1165/rcmb.2020-0360OC)	5.9
10	47	23.5	2022	Pan RY	CELL METABOLISM	Positive feedback regulation of microglial glucose metabolism by histone H4 lysine 12 lactylation in Alzheimer's disease	27.7

### 3.5 Journal publication analysis

A total of 198 papers were published in 139 academic journals. **Table 3** provides an overview of the most prominent journals contributing to this field of study. Wherein, front immunol contains the most publications (11), flowed by International Journal of Biological Sciences (5) and Science Advances (5). Among these ten journals Nature Communications had the highest IF of 14.7 in 2023, secondly Advanced Science (14.3). Moreover, the table shows that most journals belong to Q1. Among them, NATURE has the most cited articles, with a total of 192 articles, followed by CELL METAB (129 articles) and CELL (127 articles). Among these 10 journals, NATURE had the highest IF in 2023 at 50.5, followed by CELL (45.6). It is noteworthy that NATURE contains the most publications and also had highest IF (**Table 5**).

**Table 5:** The 10 top cited journal in the field of Lactylation.

Rank	Journal	Count	IF (2023)	JCR (2023)
1	NATURE	192	50.5	Q1
2	CELL METAB	129	27.7	Q1
3	CELL	127	45.6	Q1
4	GENOME BIOL	127	10.1	Q1
5	P NATL ACAD SCI USA	126	9.4	Q1
6	NAT COMMUN	125	14.7	Q1
7	CELL REP	119	7.5	Q1
8	MOL CELL	107	14.5	Q1
9	NAT METAB	94	19.2	Q1
10	FRONT IMMUNOL	93	5.7	Q1

### 3.6 Keyword analysis

All keywords were extracted from the 198 publications and subjected to a co-occurrence analysis using Citespace software in order to gain an insight into the current areas of research in the field of *lactylation*

in cancer. The top 20 high-frequency keywords are shown in **Table 6**. Apart from “expression” (63 times), the keywords that appeared most frequently (more than 20 times) were “gene expression” (53 times), “Histone lactylation” (39 times), “metabolism” (35 times), “metabolic regulation” (30 times), “lactate” (28 times), “activation” (26 times), “cancer” (24 times) and “cells”(23 times). The Citespace software was used to cluster the keywords and to construct a time line for the keywords following the clustering process (**Figure 5E**). A total of 10 clusters were created: #0bladder cancer; #1myoblast differentiation; #2 lysine lactylation; #3 hepatocellular carcinoma; #4posttranslational modification; #5lactatemmetabolism; #6immunometabolism; #7metabolic reprogramming; #8complexes; #9stem cells and#10 succinylation.

**Table 6:** Top 20 high-frequency keywords in the field of Lactylation.

Rank	Keyword	Count	Rank	Keyword	Count
1	expression	63	11	lactic acid	18
2	Gene expression	53	12	glycolysis	16
3	histone lactylation	39	13	aerobic glycolysis	12
4	metabolism	35	14	inflammation	11
5	Metabolic regulation	30	15	cell	10
6	lactate	28	16	protein	10
7	activation	26	17	monocarboxylate transporters	10
8	cancer	24	18	resistance	9
9	cells	23	19	differentiation	9
10	acetylation	19	20	gene	9

CiteSpace’s Keyword Emergence Analysis allows predicting future research frontiers. Originally proposed by Kleinberg, the method is based on a burst word monitoring algorithm. This algorithm allows the identification of burst words, which to some extent reflect research trends in the field over a given period, by accurately identifying keywords with high frequency growth over a given period. The emergence of six keywords from Burstiness in the CiteSpace software is illustrated in the following graph. Over time, the color of prominent words changes from green to red, and some words return to green. The time at which the word first appeared is indicated by the length of the red line. The six most prominent keywords in terms of emergence are shown in **Figure 5F**.

### 3.7 Burst keywords analysis

To facilitate the identification of research hotspots in this area, Citespace is used to identify the six most cited keywords with the strongest citation bursts. The largest breakout occurred in 2020, when the breakout was 1.33 and lasted 1 year. Obviously, it is an indication that research on lactylation is a hot spot in terms of differentiation. The lysine lactylation and glucose datasets, which began in 2022 and continued until the data were collected, had the longest burst duration. In addition to the aforementioned keywords, we are also interested in other potential keywords that may guide future research directions.

## 4 Discussion

Lactate, which is derived from pyruvate in tumor cells, is a well-documented source of energy and a metabolic by-product. It is worthy of note that lactylation has been demonstrated to exert a pivotal influence on the etiology of cancer and inflammation. Previously, no bibliometric analysis study had been conducted on lactylation. So, this study is the first bibliometric analysis on lactylation with a timeline from 2019 to 2024. Lactylation is an emerging and rapidly developing field. Gaining a comprehensive understanding of developments in this area has been a significant challenge.

A total of 198 publications on lactylation between 2019 and 2024 were identified in the Web of Science Core Collection database during the course of this study. The productivity of a field is largely reflected in the number of articles published in that field. Although the number of articles in this field is not very large, the increase in the number of articles has been exponential in comparison with previous years. In 2019, Zhang introduced the concept of Lactylation for the first time. After that, articles on lactylation have been springing up all over the place. There are two distinct periods in the annual growth curve: a period of steady development (2019–2021) and a period of rapid development (2022–2024). In the initial three-year period, the field garnered significant attention and became a popular phenomenon from 2022 onwards. During the period of accelerated growth, a number of significant discoveries were made, which contributed to the rapidly expanding field. The use of lactylation in cancer therapy continues to evolve and more lactylated sites continue to be discovered. There is belief that lactylation may have a miraculous role in cancer treatment in the future.

It is notable that the entirety of the top 10 research organizations is based in China, which serves to illustrate that research institutions in China are still at the vanguard of research in lactylation. The USA also has a substantial and influential presence in lactylation research. Both countries rank among the world’s leaders in terms of gross domestic product (GDP) and scientific and technological output. It is noteworthy that China commenced its research activities at an early stage and has subsequently demonstrated the greatest productivity in recent years. In addition, we found a number of publications from CAS and SJTU, institutions that have made remarkable contributions to the development of lactylation.

Furthermore, the application of keyword clustering enables the description of the knowledge structure of a given discipline, as well as the identification of its research frontiers. Cluster analysis revealed three major clusters in lactylation research, indicating the effect of lactylation in bladder cancer, myoblast differentiation via lactylation and lysine lactylation. These are the three main aspects of the lactylation issue. Clearly, lactylation plays an important role in cancer and inflammation.

In order to identify emerging research frontiers, keywords that are frequently cited over a period of time were used in a burst detection analysis. Only the top 6 keywords with the strongest citation bursts in published articles on lactylation research from Citespace are shown due to the small sample size. The blue line represents the time interval and the red line represents the duration of the quote burst. The results showed that differentiation was the most prominent burst keyword. It was first observed in 2020 with a burst strength of 1.33, followed by polarization (1.13). The remaining four keywords are metabolic regulation (0.23), inflammation (1.61), lysine lactylation (0.21) and glucose (0.21).

Nevertheless, it should be noted that the present work was not without limitations. First, the Web of Science Core Collection database was queried for this bibliometric analysis. It is possible that papers of high quality that are published in journals that are not included in the Web of Science Core Collection database may be overlooked. Secondly, the sample size of this study was relatively limited, which may have affected the reliability of the findings. Moreover, the most frequently cited keywords employed in the statistical analysis were constrained by the paucity of published literature in this domain. Thirdly, the CiteSpace and VOSviewer software has certain inherent limitations in this study. Citespace and VOSviewer may not be capable of full-text analysis of the literature and some information may have been overlooked. Finally, there is still a lot of work to do and our analysis will be subject to updates in the future.

## 5 The roles of lactylation in diseases

**Figure 6:**
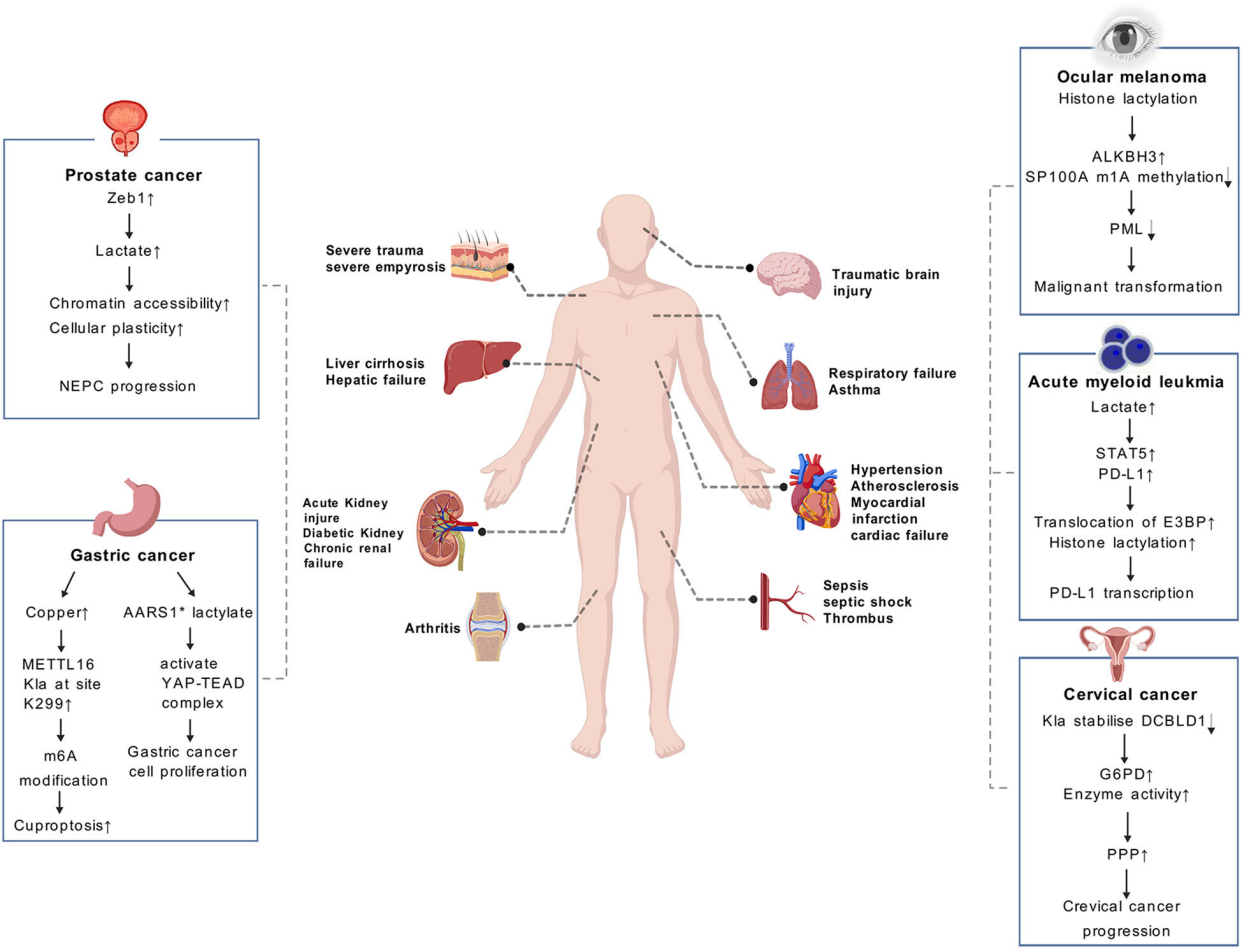
Lactate causes a number of diseases in different systems of the body. The role of lactylation (Kla) in cancer are illustrated in the boxes on both sides of the page, including prostate cancer, gastric cancer, ocular melanoma, etc. Kla plays a significant role in clinical diagnosis and prognosis of cancers. (AARS1* means AARS1 with lactyltransferase activity).

The widespread Kla involves a variety of biological processes. It is noteworthy that Kla’s disorder involves a myriad of pathological processes in almost all organs, including liver injure (18), Myocardial ischemia-reperfusion injury (17, 19), neurodegenerative diseases (20), cerebral ischemia (21), autoimmune uveitis (11, 22), pulmonary fibrosis (23, 24), acute kidney injury (25), sepsis (26) and rheumatoid arthritis (27, 28) (**Figure 6**). In this review, we aim to elucidate the impact of Kla on the pathogenesis of a variety of human diseases, including tumors, cardiovascular diseases, pulmonary diseases, diseases of the nervous system and other complex diseases. In this way, we hope to gain a more profound comprehension of the underlying mechanisms of Kla-mediated pathogenicity.

### 5.1 Lactylation in tumor

The regulation of Kla by lactate has recently been established as a novel factor influencing tumor progression, including cell proliferation, invasion and angiogenesis through affecting metabolic reprogramming and immune microenvironment of tumor (29). Enhanced glycolysis and the accumulation of lactate are commonly observed in various types of cancer, which has been found to drive a recently discovered PTM known as Kla of histones and nonhistones (6, 30).

Scientific research has demonstrated the role of histone Kla in promoting tumor progression. A recent study has shown that histone lactylation is involved in the process of in the metabolic rewiring and epigenetic reprogramming of prostate cancer, allowing the cancer to switch from adenocarcinoma to neuroendocrine. The results of this study demonstrate that Zeb1 is involved in the regulation of the expression of several key glycolytic enzymes, which consequently affects the likelihood of tumor cells utilizing glycolysis as a metabolic pathway. Subsequently, the accumulation of lactate, which is mediated by histone lactylation, enhances chromatin accessibility and cellular plasticity. This includes the induction of neuro-gene expression, which in turn promotes the development of NEPC (12). The research team, headed by He et al, discovered that a deficiency in the Numb/Parkin pathway results in a metabolic reprogramming characterized by a substantial increase in lactate acid production. This, in turn, gives rise to an elevation in histone lactylation and the transcription of neuroendocrine-associated genes in prostate or lung adenocarcinomas (13). Moreover, the involvement of histone lactylation has been suggested in the control of diverse forms of chemical alterations. For example, the results of recent research indicate that histone lactylation improves ALKBH3 expression and simultaneously attenuates the formation of tumor-suppressive promyelocytic leukemia protein (PML) condensates by removing the m1A methylation of SP100A. This process promotes the malignant transformation of ocular melanoma (31). In addition, Kla has been demonstrated to be a crucial factor for various immune cells within the TME. Gu et al. have demonstrated that lactate accumulation in bone marrow was positively related to STAT5 as well as PD-L1 expression in newly diagnosed AML patients (14). Increased lactate accumulation promotes nuclear translocation of E3BP and facilitates histone lactylation, which ultimately induces PD-L1 transcription in acute myeloid leukemia cells. Consequently, AML patients with STAT5-induced exuberant glycolysis and lactate accumulation may potentially benefit from PD-1/PD-L1-based immunotherapy (14).

In addition to histone modifications, Kla has also been found to occur on non-histone proteins, which have been shown to promote tumor development. Yang et al. found that Kla plays a crucial role in regulating cellular metabolism and may enhance hepatocellular carcinoma (HCC) progression (6). By conducting an integrative analysis of the lactylome and proteome, it was determined that there are a total of 9,275 Kla sites, including 9,256 sites on non-histone proteins. This suggests that Kla is a pervasive modification that extends beyond histone and transcriptional regulation (6). The research additionally revealed that Kla exerts a preferential influence on enzymes engaged in metabolic pathways, encompassing the tricarboxylic acid cycle, carbohydrate, amino acid, fatty acid and nucleotide metabolism. This consequently contributes to the advancement of HCC (6). A recent study has shown that the concentration of copper is markedly elevated in gastric cancer (GC). It has been shown that copper stress promotes METTL16 lactylation at site K229, which subsequently leads to cuproptosis via m6A modification on FDX1 mRNA in gastric cancer (30). Ju et al. demonstrated that AARS1 with lactyltransferase activity is capable of sensing intracellular lactate and translocating into the nucleus to lactylate and activate the YAP-TEAD complex, thereby promoting gastric cancer (GC) cell proliferation (15). Furthermore, lactylation has been demonstrated to stabilize DCBLD1, which has been shown to primarily stimulate PPP by upregulating glucose-6-phosphate dehydrogenase (G6PD) expression and enzyme activity, thereby promoting cervical cancer progression (32).

### 5.2 Lactylation in cardiovascular disease

With an ageing population, the prevalence and mortality of cardiovascular disease (CVD) are increasing year by year, which threatens human health and has a major influence on the quality of life and survival of patients. The role of carat in cardiovascular disease has received increasing attention in recent years. Zhang et al. demonstrated that lactate efflux-associated monocarboxylate transporter 4 (MCT4)-mediated histone lactylation is strongly associated with atherosclerosis. Furthermore, they showed that macrophage MCT4 deficiency favors the amelioration of atherosclerosis. From a mechanistic perspective, histone lactylation plays a pivotal role during the stage of specific local repair process that M1 to M2 transformation. Dependent on MCT4 deficiency, histone H3 lysine 18 lactylation has been observed to activate the transcription of anti-inflammatory genes and tricarboxylic acid cycle genes, thereby initiating local repair and homeostasis (29). Wang et al. put forth the hypothesis that histone lactylation plays a pivotal role in regulating the anti-inflammatory and pro-angiogenic activities of monocyte-macrophages, whereby it facilitates reparative gene transcription. Their findings also confirm that histone lactylation contributes to the establishment of a reparative environment and improves cardiac function following myocardial infarction (30). A study which is published recently found that α-myosin heavy chain (α-MHC) K1897 lactylation is a significant determinant of overall cardiac structure and function. Mechanistically, excessive Lactate Efflux and Depletion in Cardiomyocytes May Reduce Intracellular Lactate Levels and Thus Reduce α-MHC K1897 lactylation During Myocardial Injury (6). Moreover, Yu et al. demonstrated that HSPA12A enhances Smurf1-mediated Hif1α protein stability, thereby increasing glycolytic gene expression and maintaining optimal aerobic glycolytic activity to sustain H3 lactylation during reperfusion. This improves cardiomyocyte survival and attenuates myocardial ischemia/reperfusion injury in the final analysis (11).

### 5.3 Lactylation in pulmonary disease

Chronic pulmonary disease represents a significant global health concern, with a considerable social and economic impact (33). Recent study found that H3K18 lactylation promotes the progression of arsenite-related idiopathic pulmonary fibrosis (As-IPF) (24). From a mechanistic perspective, Elevated overall levels of m6A, YTHDF1 and m6A-modified neuronal protein 3.1 (NREP) were observed in alveolar epithelial cells (AECs). Increased TGF-β1 secretion through the YTHDF1/m6A/NREP pathway, which promotes fibroblast-to-myofibroblast transformation (FMT) (24). Recently, Chen et al. demonstrated that H3K18 lactoylation driven by mROS-mediated glycolytic shifts plays a pivotal role in the development of hypoxic pulmonary hypertension (34). A recent study has shown that glycolysis and glycolysis-dependent protein lactylation are significantly elevated in the lungs of asthmatic mice and OVA-stimulated THP-1 cells. The aforementioned effects were found to be inhibited by dexamethasone (DEX). The findings of this study may offer a novel approach to the management of eosinophilic asthma (35).

### 5.4 Lactylation in diseases of the nervous system

There is growing evidence that lactic acid is produced during brain glycolysis, and changes in lactic acid may be associated with neuropsychiatric disorders. Wang et al. have demonstrated that impaired brain glucose metabolism is an early indicator of Alzheimer’s disease (AD). Furthermore, the level of isocitrate dehydrogenase 3β (IDH3β), a key tricarboxylic acid cycle enzyme, has been found to be significantly decreased in the brains of AD patients and AD transgenic mice (36). The knockdown of IDH3β has been demonstrated to induce oxidation-phosphorylation uncoupling and promote histone lactylation, thereby enhancing the expression of the paired-box gene 6 (PAX6). The elevated PAX6 in turn inhibited the IDH3β expression, which resulted in tau hyperphosphorylation, synapse impairment, and learning and memory deficits that were similar to those observed in AD. This ultimately led to the formation of a positive feedback inhibitory loop. Therefore, exploring interrupting this positive feedback inhibitory loop by up-regulating IDH3 β or down-regulating PAX6 might attenuate AD neurodegeneration and cognitive impairment (36). The etiology of schizophrenia (SCZ) is multifactorial, with both genetic and environmental factors contributing to its development. A recent study has identified disruption of lactate signaling between astrocytes and neurons as a potential mechanism underlying SCZ (37).Its pathogenesis may involve altered lactylation modification (38). Furthermore, lactylation may represent a novel posttranslational modification in the context of inflammation in neonatal hypoxic-ischemic encephalopathy (39). A study indicated that cGAS deficiency regulates the phenotypic polarization and glycolysis of microglia through lactylation in a hypoxic-ischemic encephalopathy cell model (40).

### 5.5 Lactylation in liver diseases

Various studies have identified the engagement of lactylation in the pathogenesis of liver diseases, particularly in the context of liver fibrosis, liver injury and non-alcoholic fatty liver disease (NAFLD). RNA-seq and CUT&Tag staining mass spectrometry analyses showed that induction of hexokinase 2 (HK2) expression in activated hematopoietic stem cells is a prerequisite for histone lactylation gene expression. Furthermore, the fate of the HSC is determined by histone lactylation via the production of lactate by activated HSCs. The results of this study indicate that HK2 may represent a viable therapeutic target for the treatment of liver fibrosis (41). Li et al. demonstrated that lactate released from the microenvironment of acetaminophen-induced acute liver injury elevated Caspase-11 levels, facilitated gasdermin D activation, and accelerated macrophage pyroptosis. These findings suggest that these processes contribute to the exacerbation of liver injury. Further experiments demonstrated that NEDD4 K33 lactylation inhibits protein interactions between Caspase-11 and NEDD4, thereby promoting APAP-induced liver injury through Caspase11-dependent non-canonical pyroptosis (42). Gao et al. discovered that mitochondrial pyruvate carrier 1 (MPC1) regulates the lactylation of fatty acid synthase and plays a role in the treatment of non-alcoholic fatty liver disease (43).

### 5.6 Lactylation in Ischemia-reperfusion injury

Ischemia-reperfusion injury (IRI) is a critical pathological process that significantly threatens patient safety. Lactate production increases during IRI, making lactate a valuable indicator for assessing IRI severity. In myocardial IRI (MI/RI), Fang et al. demonstrated the key role of lysine lactylation (Kla) in MI/RI pathogenesis. Their findings indicate that lactate dehydrogenase A (LDHA) promotes MI/RI by enhancing NLR family pyrin domain containing 3 (NLRP3) inflammasome Kla. This modification stabilizes NLRP3 and induces cardiomyocyte pyroptosis (44). Furthermore, in liver IRI, Du et al. discovered that heat shock protein A12A (HSPA12A) reduces lactate production during glycolysis. Consequently, HSPA12A inhibits the lactylation of high-mobility group box 1 (HMGB1), suppresses exosome secretion, and diminishes macrophage activation, chemotaxis, and associated inflammatory cascades, thereby conferring a protective effect on hepatocytes (18). These findings suggest HSPA12A as a potential therapeutic target for hepatic IRI (HIRI).

Finally, in cerebral IRI, Lopez et al. observed that increased genome-wide histone H3K18 lactylation levels promote the conversion of T cells from a pro-inflammatory phenotype to a regulatory T cell (Treg) phenotype (45). Protein lactylation can enhance the expression of anti-inflammatory cytokines IL-10 and TGF-β, contributing to reduced inflammation. This modification also regulates macrophage polarization, promoting the M2 phenotype while reducing the proportion of pro-inflammatory M1 macrophages, thereby mitigating the inflammatory response (46). In mouse models of cerebral IRI, researchers modulated lactylation levels to investigate its effects on antioxidant enzyme activity and oxidative damage. Increased protein lactylation significantly enhanced superoxide dismutase (SOD) and catalase (CAT) activities while reducing reactive oxygen species (ROS) levels and oxidative damage in brain tissue (47, 48). Additionally, protein lactylation modification can reduce mitochondrial membrane permeability, prevent cytochrome c release, and inhibit apoptotic signaling by regulating Bcl-2 family proteins (49).

### 5.7 Lactylation in other complex diseases

In recent years, lactylation has been identified as a potential factor in the etiology of a number of diseases. Huang et al. demonstrated that YY1 lactylation plays a role in enhancing activation of microglia and promotion of their proliferation and migration capacity in autoimmune uveitis (11). From a mechanistic perspective, YY1 lactylation was found to promote microglial dysfunction in autoimmune uveitis, with this effect being mediated by the upregulation of inflammatory cytokine secretion and the promotion of cell migration and proliferation. The findings indicated that the treatment of autoimmune uveitis may be efficacious when targeting the lactate/p300/YY1 lactylation/inflammatory genes axis (11). It was demonstrated by An et al. that pyruvate dehydrogenase E1 component subunit alpha (PDHA1) hyperacetylation and inactivation enhance lactate overproduction, which in turn mediates fission 1 protein (Fis1) lactylation and exacerbates sepsis-induced acute kidney injury (SAKI). The reduction of lactate levels and Fis1 lactylation has been demonstrated to attenuate SAKI (25).

### 5.8 Disease therapy targeting lactylation

Kla regulation has been identified as a principal avenue of treatment for a diverse array of diseases. Targeted lactylation involves the production and transport of lactate and other lactate metabolic processes and has attracted extensive attention from clinical researchers. Currently, the diagnostic and therapeutic targets associated with Kla has witnessed more substantial progress (50). LDHA, which catalyzes the reduction of pyruvate to lactate, is highly expressed in various tumor tissues and has been associated with poor prognostic outcomes in cancer patients (51). Researchers have identified a number of highly efficacious LDH inhibitors, including Oxamate, which has been shown to inhibit lactate production and lactylation modifications, thereby blocking the downstream pathway of lactylation. A number of these inhibitors have progressed to phase I and phase II clinical trials (21, 52, 53). Studies have recorded that the application of oxamate to lymphoma cells hinders the advancement of acute lymphoblastic leukemia cell (54). Oxamate also has been observed to hinder the growth of cells in nasopharyngeal and gastric cancer (55). Gossypol, FX11 and quinoline 3‐sulphonamides are part of the group of LDHA inhibitors that compete with nicotinamide adenine dinucleotide. Several research studies have extensively recorded the effectiveness of these in inhibiting the proliferation of different types of cancer cells, such as those found in the colon, neuroblastoma, HCC, primary pancreatic cancer, melanoma and breast cancer (55).

In addition, monocarboxylate transporters (MCTs), carrying out H+-lactate export, were found overexpressed and contribute to the acidity of the tumor microenvironment and has been demonstrated to correlate with prognosis. Therefore, MCTs is considered attractive targets, especially MCT1 and MCT4 have been reported to promote elevated lactate levels in a variety of diseases including cancer, idiopathic pulmonary fibrosis, and myocardial infarction (24, 56, 57). Anna et al. demonstrated that partial MCT1 invalidation offers protection against diet-induced non-alcoholic fatty liver disease and the associated brain dysfunction. This finding represents a new preventive or therapeutic target (58). Qian et al. suggested that MCT4-dependent lactate secretion suppresses antitumor immunity in LKB1-deficient lung adenocarcinoma (59). Numerous MCT blockers have exhibited effectiveness in preclinical experiments. MCT inhibitors derived from natural products, such as quercetin and phloretin have been effectively treated in various malignancies, such as lung cancer, esophageal cancer, breast cancer and other types of cancer (60). Along with natural products, synthetic molecules, such as lonidamine, DIDS, simvastatin and other MCT inhibitors have exhibited therapeutic potential for a range of cancer types (60, 61).

## 6 Conclusions and prospects

A bibliometric analysis was conducted on the literature on lactylation published in the last few years. What we found was that the field of lactylation is growing rapidly as research in the field continues to increase, with China assuming a prominent position of leadership in this field. In the early stages of study, Scholars have concentrated their research efforts on elucidating the role of lactylation modifications, which are induced by lactate metabolism, in the etiology of disease. The current focus of research in this field is the exploration of the role of lactylation modification sites and the specific mechanism mediated by lactylation modification in a wide range of diseases. In the future, disease intervention strategies targeting lactylation regulatory networks can be explored from multiple perspectives, spanning fundamental research to clinical translation. Firstly, we could analyze the cell-specific mechanisms of lactylation in the immune-metabolic microenvironment, Key questions include: how lactylation shaped the tumor-promoting microenvironment in different immune cells (TAMs, T cells, B cells) and tumor cells? How does the modification site dynamics respond to metabolite gradients? Combined analysis of cell type-specific lactatomics and spatial metabolomics was developed to map the modification in tumor/inflammatory lesions, so as to elucidate the mechanism of lactatation-mediated epigenetic regulation of tumor progression by immune checkpoint molecules (PD-L1, CTLA-4) and provide new targets for combined immunotherapy. Secondly, To explore the function role of non-histone protein lactylation in signaling pathway dysregulation, Research is needed to determine how lactylation modifies key signaling molecules—including transcription factors (e.g., p53, HIF-1α), kinases (e.g., mTOR, AMPK), and metabolic enzymes (e.g., PKM2, LDH)—to drive disease phenotypes. Thirdly, can lactylation biosensors designed with gene-encoded probes based on FRET monitor the dynamics of lactylation *in vivo* in real time, facilitating “visualized epigenetic therapy”? High-throughput screening of allosteric inhibitors of lactylation targeting the Kla domain of p300/CBP or development of PROTAC degradators targeting lactylation to explore precise intervention strategies. Finally, targeted lactylation therapy is explored, such as nano-delivery of lactatation inhibitors to specific tissues. There is an increased emphasis on targeted therapy for tumors and other related diseases.

Although there is mounting evidence that lactylation represents a promising therapeutic target to inhibit disease progression, the exact mechanism through which this occurs remains unclear. It is therefore imperative to pursue further investigation into the identification of the specific “writers”, “erasers” and “readers” of lactylation modification, in order to facilitate more precise targeting of lactylation and to provide new potential targets for tumor therapy. This study highlights the crucial direction for future research in the field of lactylation modification, and offers new insights for the study, diagnosis and treatment of disease mechanisms.

## Data Availability

The raw data supporting the conclusions of this article will be made available by the authors, without undue reservation.
